# The Lightweight Design of a Seismic Low-Yield-Strength Steel Shear Panel Damper

**DOI:** 10.3390/ma9060424

**Published:** 2016-05-27

**Authors:** Chaofeng Zhang, Jiajia Zhu, Meiping Wu, Jinhu Yu, Junhua Zhao

**Affiliations:** Jiangsu Key Laboratory of Advanced Food Manufacturing Equipment & Technology, Mechanical Engineering School of Jiangnan University, Wuxi 214122, China; zcf830703@163.com (C.Z.); hebeizhujj@163.com (J.Z.); jinghutiger@163.com (J.Y.); junhua.zhao@163.com (J.Z.)

**Keywords:** seismic engineering, low-yield-strength steel, shear panel damper, lightweight design, material property, boundary constraints

## Abstract

The lightweight design and miniaturization of metallic dampers have broad application prospects in seismic engineering. In this study, the superplastic property and the maximum energy dissipation capacity per unit mass of low-yield-strength steel (LYS) are investigated via comparison with those of several common metallic damping materials by tests. Additionally, the boundary constraints of an LYS shear panel damper are studied further. Our experimental results suggest that LYS is an excellent damping material for achieving the lightweight design goal. A novel design of a lightweight damper, having excellent deformation ability and robust mechanical properties, is presented. The findings of this study are expected to be useful in understanding the lightweight design of dampers.

## 1. Introduction

In recent years, several large earthquakes have occurred worldwide. With the aim of mitigating the effects of earthquake disasters, both active and passive dampers have been widely adopted as energy absorption devices in civil engineering. In particular, passive dampers play a key role on account of their technical simplicity and wide applicability. A metallic damper is preferred in practical engineering owing to its high reliability and good low-cycle fatigue properties. Metallic dampers can be used to absorb energy by reciprocal bending [1,2,3], tension-compression [4,5,6], and shear [7,8] methods, as well as by various combinations of these three methods. Since a shear panel damper (SPD) has increasingly been used in seismic structures, it was chosen as the research focus of this study.

Considerable efforts have been devoted toward investigating the responses of various structures fixed with a metallic SPD when the structures are subjected to nonstationary earthquake ground motion [9,10]. The effective damping ratio [11,12], effective stiffness [13,14], and hysteretic curve [15] are important factors in time history analysis. Meanwhile, buckling [16,17,18,19] is generally considered an important factor affecting the stability and the hysteretic performance of the damper. Several different types of SPDs have been developed and optimized for achieving a good hysteretic curve and good fatigue performance [20,21,22].

The lightweight design of a damper is crucial for building construction. If metallic dampers are not designed to be lightweight, the damper weight often needs to be re-evaluated in the load-bearing design of a building. Furthermore, installation and replacement of a heavy damper must be carried out with the aid of lifting equipment, which leads to a sharp increase in installation cost and the inconvenience of replacement. Therefore, research on decreasing the weight of metallic dampers and their miniaturization is of great significance.

Generally, lightweight [23,24] or high-ductility materials are selected for designing lightweight SPDs. However, it is also necessary to determine which material is more suitable for lightweight design. Owing to the high deformation capacity of pure aluminum [24] and low-yield-strength steels (LYS’s), both are suitable materials for lightweight design. In the present study, LYS’s were selected as the damper materials. To achieve the lightweight design goal, it is also necessary to design the corresponding damping structure by extracting the maximum material performance under the loading condition. In addition, the damping force, deformation capacity, and energy dissipation characteristics of dampers should simultaneously meet the usage and safety requirements of seismic engineering. Here, the lightweight design and mechanical performance of an LYS shear panel damper (LYSPD) are discussed in detail.

The in-plane deformation of the LYSPD is considerably stable; therefore, it possesses a high dissipation capacity and can be used as a lightweight damper. Several effective strategies are available for mitigating buckling phenomena. The out-of-plane displacement can be prevented by using a buckling inhibited panel [25,26] and delayed by using transverse or cross stiffeners [27]. The deformation capacity of the SPD can be improved by these two methods, but the improvement is not notable. In addition, these two methods do not satisfactorily address the goal of miniaturization of the SPD.

In a previous work [20], it was experimentally proved that both the goals of lightweight design and miniaturization of the SPD could be achieved simultaneously by using a reasonable design of the shape and size of the unstiffened shear panel. In an incremental cyclic test, stable in-plane deformation was observed before the occurrence of cracking. Based on this previous work [20], in the present study, the boundary constraints (such as the stiffener, transition arc, and rib) on the LYSPD were further studied via tests. Boundary constraints play a key role in the large shear plastic deformation and stability of LYSPDs. The findings of the present study are expected to be highly useful for understanding the lightweight design of dampers.

## 2. Test Procedures

### 2.1. Material Property Test

Normally, the ultimate bending or shear characteristics of a material under large plastic deformation cannot be predicted or well described by the theoretical conversion of coupon test results. Therefore, both the ultimate material performance and the maximum energy dissipation capacity of the damper need to be directly verified by experiments. Since the ultimate energy dissipation capacity of materials depends on the loading conditions, a key issue of the lightweight design is that the damper structures need to be formalized according to their bearing loads. However, few studies have systematically compared the ultimate material performance of damping materials under different loading conditions by tests.

In this study, several metallic damping materials, such as aluminum 6061 (AL6061), carbon steel (Q235), and stainless steel 316 (SS316), are tested. Owing to their high plastic deformation capacity, LYS’s have been widely applied in seismic engineering. Here, three types of LYS’s—LYS225, LYS160, and LYS100, with yield strengths of 225 MPa, 160 MPa, and 100 MPa, respectively—are also tested via comparison with the abovementioned metallic damping materials. The geometric sizes of all the test specimens of all the materials are listed in Table 1.

**Figure 1:**
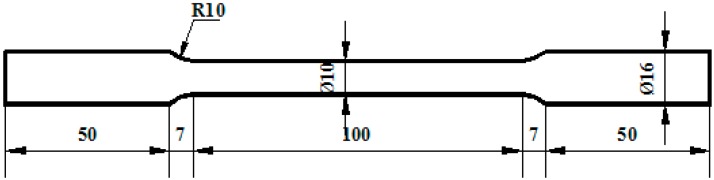
Geometry of round bar specimen (mm).

Typical round bar specimens are employed in all of the tensile and torsion tests (see Figure 1). The effective diameter and length of the bars are 10 mm and 100 mm, respectively. Six specimens of each material are employed, of which three are used for the tensile tests and three are used for the torsion tests.

### 2.2. The Lightweight Design of Shear Panel Damper

#### 2.2.1. Concept of Lightweight Design

Accumulative dissipation energy of the damper is crucial in seismic design and is calculated as follows:

(1) $$E=\sum_{i=0}^{N}F_{i}S_{i}$$

where *E* is the accumulative dissipation energy; *F* and *S* are the damper force and the damper displacement, respectively, in each loading step; and *N* is the step number. When the damper force is established according to the structural seismic design and there is no out-of-plane buckling, the energy dissipation capacity of the damper depends on the displacement; therefore, the high deformation capacity of the damper is the sole key parameter for the lightweight design.

(1) Lightweight Design of Boundary Constraints

**Figure 2:**
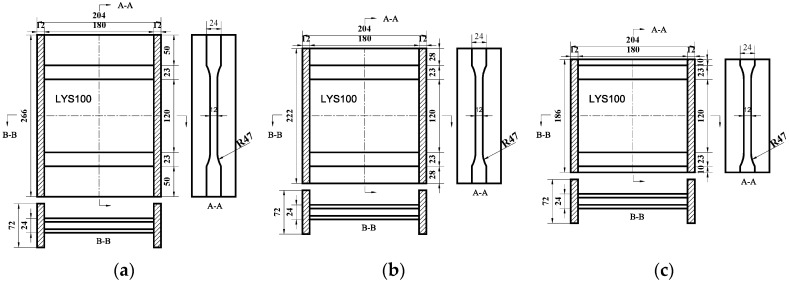

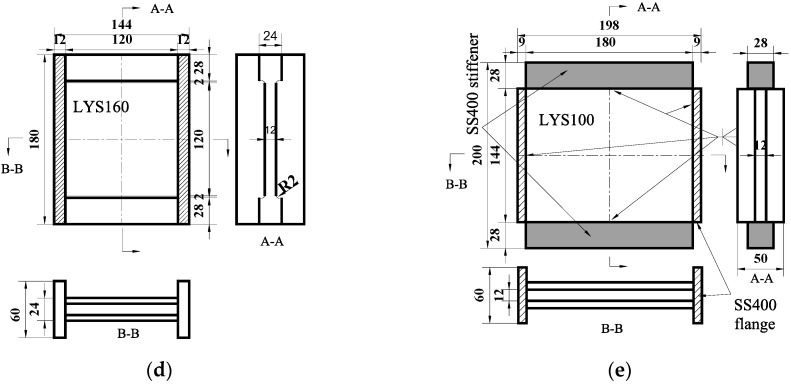
Damper shape: (**a**) SPD100-50; (**b**) SPD100-28; (**c**) SPD100-10; (**d**) SPD160-28; and (**e**) SPD100-rib (mm).

Based on the present tests, it is known that LYS’s possess both high deformation capacity and the maximum dissipation energy under shear deformation, which suggests that the LYSPD should be the optimal selection for the lightweight design. An LYSPD made from LYS100 with high deformation capacity was developed in a previous study [20]. Stiffeners and transition arcs were employed on the upper and lower sides of the shear panel to prevent the overlap of the plastic hinge and welding seams. Ribs were introduced on the left and right sides of the shear panel to alleviate the corner stress concentration. Adoption of these perfect damper boundary constraints is the precondition for restraining the out-of-plane buckling of the shear panel, and it ensures large plastic deformation. However, the weight of these boundary constraints accounts for 82% of the total weight of the damper (see Figure 2a). Therefore, it is necessary to optimize the lightweight design of the boundary constraints first.

(a) Stiffener

The LYSPD becomes brittle in the heat-affected zone [28] owing to the welding stiffener, which will lead to a decrease in the damper deformation capacity. The width of the heat-affected zone is usually around 8–12 mm. The stiffener height is set to four times the width of the heat-affected zone in SPD100-50 (see Figure 2a). This leads to an excessive safety margin and increases the weight of the damper. To reduce the weight, the stiffener height of SPD100-28 is set to two times the width of the heat-affected zone (see Figure 2b). In consideration of the transition arc, a small margin is left in the stiffener height of SPD100-10 (see Figure 2c). On the basis of the test results, the optimal stiffener height is presented and discussed later.

(b) Transition Arc

A sharp change in a section in the structure will lead to stress concentration and easy failure. In order to prevent stress concentration, the transition arc is generally arranged along the stress direction, as was done in the tensile test specimens in this study (see Figure 1). However, the large transition arc between the shear panel and the stiffeners may not have been necessary in the previous design of the LYSPD. Because the stress along the transition arc does not dominate the in-plane shear deformation, the transition arc may be a redundant component and can be neglected in the design. In our tests, the performance of SPD160-28 was tested with the transition arcs removed (see Figure 2d).

(c) Rib Material

Two typical materials are adopted in current LYSPDs: LYS and ordinary carbon steel. Owing to the high rigidity of ordinary carbon steel, the stress concentration can be restrained by its thinner panel, which is advantageous for the lightweight design. On the contrary, the poorer ductility of ordinary carbon steel is likely to weaken the deformation ability of the LYSPD. A rib made from ordinary carbon steel is designed for verifying the feasibility of the lightweight design (see Figure 2e).

(2) Generality of Lightweight Design

The high deformation capacity of the LYSPD made from LYS100 was verified by tests in the previous study. With a change in the yield strength and ductility, it is still unclear whether a perfect performance of the LYSPD can be obtained with LYS160. Therefore, LYS160 is selected as the damper material for SPD160-28 (see Figure 2d). The generality of the design method of a lightweight damper structure aimed at achieving large plastic deformation can also be identified by tests.

(3) Shear Force and Lightweight Design

A sufficient amount of damper force is needed to absorb the seismic energy; however, the increase in the panel strength is not always positive for a system to be used as a damper. The maximum shear force of an SPD is set lower than the structural yield force in order to prevent failure of the main structure. For achieving a lightweight design, it is preferred that a larger force be provided by a smaller damper.

Several methods are available for increasing the damper force, such as increasing the number of dampers, increasing the thickness of a damper, or increasing its width. The latter two methods are advantageous for the lightweight design. However, the thickness and width of the panel are also important factors affecting the failure mode of the panel. These factors are of high relevance to the deformation capacity of the LYSPD. It is well known that the stability of the shear panel is given by the following equation:

(2) $$R=\frac{b}{t}\sqrt{\frac{12\left(1-v^{2}\right)\tau_{y}}{k\pi^{2}E}}$$

where *E* is the modulus of elasticity (=206 × 10^3^ N/mm^2^); ν is the Poisson’s ratio (=0.3); and *t* and *b* are the thickness and width, respectively, of the shear panel. The shear buckling coefficient *k* can be expressed as

(3) $$k=5.35+4.0\times {\left(\frac{b}{a}\right)}^{2}\;\;\;\;\;\;(\frac{a}{b}>1)$$

(4) $$k=5.35\times {\left(\frac{b}{a}\right)}^{2}+4\;\;\;\;\;\;\left(\frac{a}{b}\leq 1\right)$$

where *a* is the length of the panel.

Equations (2)–(4) are generally adopted for the stability analyses of current LYSPDs. However, the significant inaccuracy of these equations arises largely because the transverse shear deformation effects are neglected. Since the transverse shear deformation effects become more important when the thickness of the panels increases, both incremental theory [29] and deformation theory [30] are adopted for describing the plastic stability accurately. In these theories, the geometric parameters and boundary conditions of the plate are still the fundamental key factors for shear buckling analysis. Furthermore, neither the stress concentration nor the plastic stability of the LYSPD under cyclic loading can be evaluated effectively. It is necessary to directly investigate the relation between the deformation capacity and the buckling or failure mode of the LYSPD experimentally.

When the panel length and width are fixed, an increase in the panel thickness will improve the panel stability. However, the corner stress concentration is a graver issue, and thus leads to a decrease in the deformation capacity of the LYSPD [31]. There are some limitations to adjusting the damper bearing force solely by the panel thickness, while an effective way to adjust the damper bearing force is to adjust the shear force by changing the panel width. To avoid out-of-plane buckling, the geometric parameters of the panel are selected from a previous work [20]. The panel height and thickness are fixed at 120 mm and 12 mm, respectively. Two different panel widths—180 mm and 120 mm—are designed for SPD100-50 and SPD160-28 respectively. The plastic stability, shear force adjustability, deformation capacity, and boundary constraint reliability of these LYSPDs are investigated. According to the optimized boundary constraints, our subsequent work will discuss, in detail, theoretical and experimental works on plastic stability, with a focus on the panel sizes.

#### 2.2.2. Test Setup

**Figure 3:**
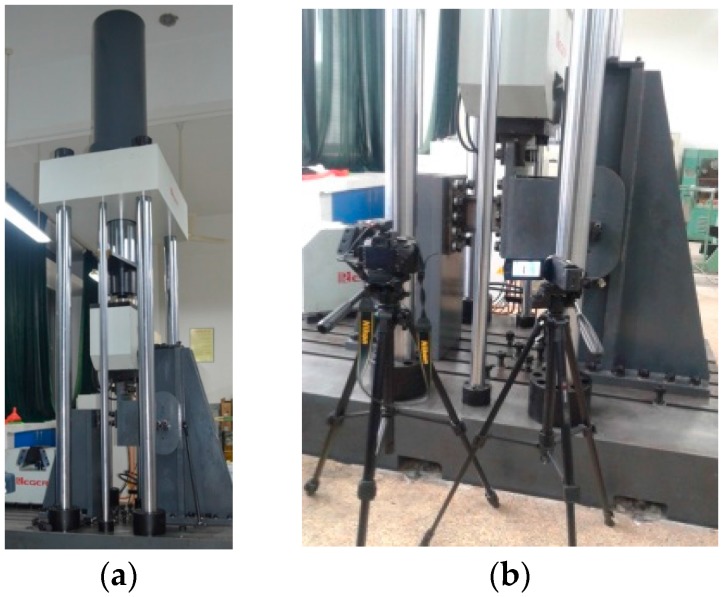
Machine for coupon and structural tests: (**a**) Before test; and (**b**) During test.

The experiments are performed in a 100-t universal testing machine (see Figure 3). The top hydraulic fixture is fixed on the 100-t actuator, which can move vertically. Different experimental procedures can be implemented simply by replacement of the bottom hydraulic fixture. When the bottom hydraulic fixture is fixed on the base, coupon or tension-compression experiments can be performed. On the other hand, shear experiments can be performed by using the shear fixture. Two parts of the shear fixture are fixed on the base, whereas one of the slide blocks of the shear fixture is connected to the actuator. The slide block can only provide vertical freedom when two groups of its rolling bearings are constrained in the vertical track. Thus, the shear test can be performed by inducing relative movement of the specimen’s two ends.

Since the damper is expected to absorb seismic energy, hysteretic incremental quasi-static loading with a control speed of 0.5 mm/s and an incremental shear strain (horizontal displacement/height) loading sequence of ±5% were applied in this study. The tests were suspended when the force decreased to 90% of the maximum or when a fatal crack appeared.

## 3. Test Results and Discussion

### 3.1. Material Test Results and Discussion

**Figure 4:**
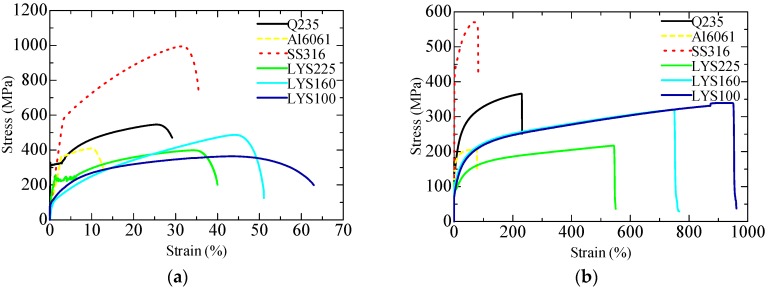
Stress–strain curves: (**a**) Tension test results; and (**b**) Torsion test results.

On account of the good consistency between the results of the tension and torsion tests, one test result for each material is selected as the representative curve to compare their basic material properties. The tensile test results are shown in Figure 4a; here, the horizontal axis represents tensile strain, and the vertical axis represents tensile stress. All LYS’s exhibited low yield strength and large plastic deformation. Although the density of Al6061 is the lowest, its ductility capacity is the poorest. The SS316 specimen exhibits both a larger stress and a larger strain than the Q235 specimen. The torsion test results are shown in Figure 4b. The shear stresses of all the materials are found to be smaller than their tensile stresses, whereas the shear strains of all the materials are found to be larger than their tensile strains. Severe plastic deformation of the LYS is observed for the first time, especially for LYS100; its shear strain is approximately 1000%. Meanwhile, deformation degradation is also observed in SS31; its ductility is higher than that of Q235 in the tension tests, whereas its ductility is rarely lower than that of Q235 in the torsion tests. Considering that not much of a difference is observed between Q235 and LYS225, there is no major advantage to using LYS225 for achieving a lightweight design.

**Figure 5:**
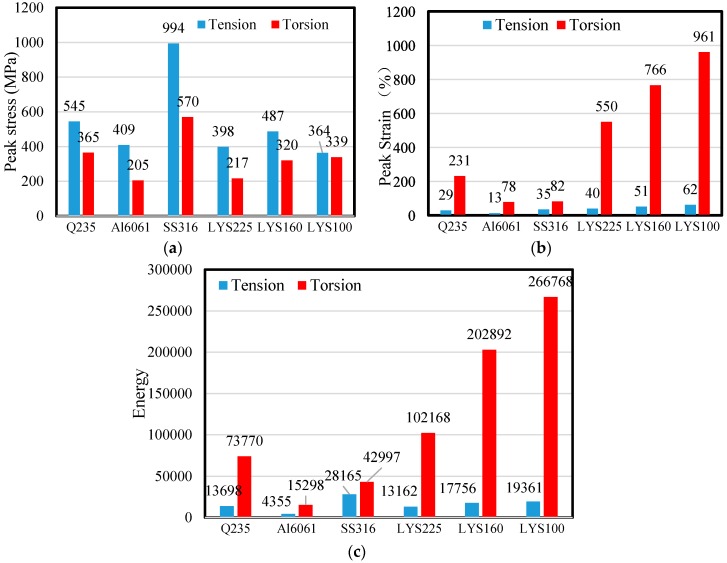
Comparison of basic properties of materials. (**a**) Peak stress comparison; (**b**) Ultimate strain comparison; and (**c**) Energy absorption comparison.

The peak tensile stress and shear stress of SS316 are 994 and 570 MPa, respectively (see Figure 5a). Both values are largest in the tension and torsion tests. Not much of a difference is observed in the tensile stress or shear stress of the remaining materials. The shear stress of Al6061 is the smallest and is only approximately 205 MPa.

Both AL6061 and SS316 exhibit the poorest shear deformation, whereas LYS100 exhibits the highest deformation capacity (peak tensile strain of 61% and peak shear strain of 961%) among all the materials (see Figure 5b). The peak strain of LYS100 is around 2 times and 4.2 times that of ordinary carbon steel under uniaxial tension and under shear, respectively. In particular, the peak shear strain of LYS100 is around 16 times its peak tension strain. These results show that LYS100 undergoes ultra-plastic deformation under shear. This phenomenon has thus far never been observed by researchers. In addition, in conventional approaches, the shear performance of materials is typically evaluated indirectly according to the coupon test results. However, the dissipation ability of LYS’s under shear could be greatly underestimated by such conventional approaches.

To meet the seismic performance requirement of structures, high-energy dissipation capacity of the damper is essential. Therefore, the ideal material for the lightweight design of dampers should exhibit both large peak stresses and large peak strains simultaneously. However, the tradeoff between high strength and low ductility has thus far not been achieved satisfactorily in material development. When the strength of the material is high, its ductility is low, and *vice versa*. Hence, it is necessary to experimentally evaluate the overall energy dissipation capacity of the material for achieving the lightweight design. From the test results, it is seen that the peak tensile stress of each material is generally no more than two times its peak shear stress. However, the peak shear strain is around two times the peak tensile strain (see Figure 5a,b). The amount of stress-strain energy (SSE) is calculated as follows:

(5) $$\mathrm{SSE}=\frac{\sum_{i=0}^{N}\left(Stress_{i}+Stress_{i+1}\right)\left(Strain_{i+1}-Strain_{i}\right)}{2}$$

where *N* is the loading step number.

The total SSE of the materials is shown in Figure 5c. The maximum SSE under uniaxial tension (SS316, 28,165 MPa·%) is generally smaller than that of other materials under shear, the exception being Al6061. This suggests that more energy can be dissipated by shear deformation. Hence, the shear structure is a preferred type of structure for the lightweight design of the damper.

The maximum SSE of AL6061 is 15,298 MPa·%, which is only around 1/17 times that of LYS100 (26,6778 MPa·%). Although the density of AL6061 (2.7 kg/m³) is around 1/3 times that of LYS100 (7.8 kg/m³), LYS100 is more advantageous for the lightweight design from the viewpoint of energy consumption per unit mass.

Owing to the ultra-high shear deformation capacity of LYS100, its SSE displays the absolute advantage (see Figure 5b,c). Moreover, the shear force can be transformed into axial force by ingenious design. The lightweight design of the braces is also feasible. On the basis of the above analysis, the LYS and shear structure are selected for achieving the lightweight design and miniaturization goals.

### 3.2. Structural Test Results

#### 3.2.1. Hysteretic Curve

**Figure 6:**
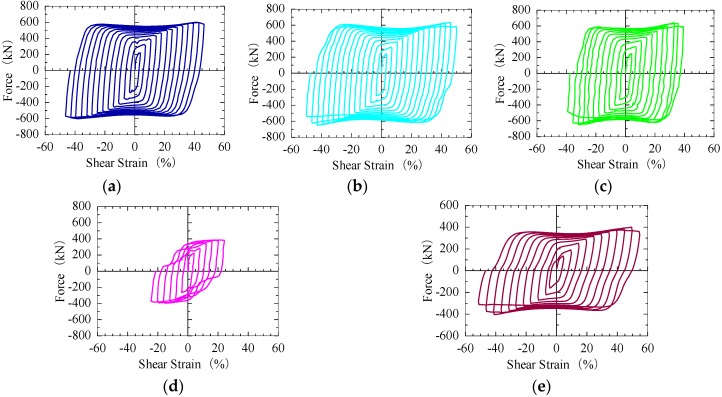
Force–shear strain curves: (**a**) SPD100-50; (**b**) SPD100-28; (**c**) SPD100-10; (**d**) SPD100-rib; and (**e**) SPD160-28.

According to the design concept, cyclic incremental tests are performed, and the test results are as shown in Figure 6. Although some pinching affects the hysteretic cycles of SPDs with LYS ribs, the hysteretic curves are approximately rectangular. This implies that the buckling is not obvious. The hysteretic curve of the SPD-rib specimen, which is peanut shaped, is quite different from those of the other material specimens. Because the rib of the SPD100-rib specimen is the only feature differentiating this specimen from the other four specimens, it can be inferred that the approximate elastic-plastic behavior of the SPD is destroyed by the rib. The deformation capacity of SPD100-rib is the poorest, which is no more than half of that of the remaining dampers.

**Figure 7:**
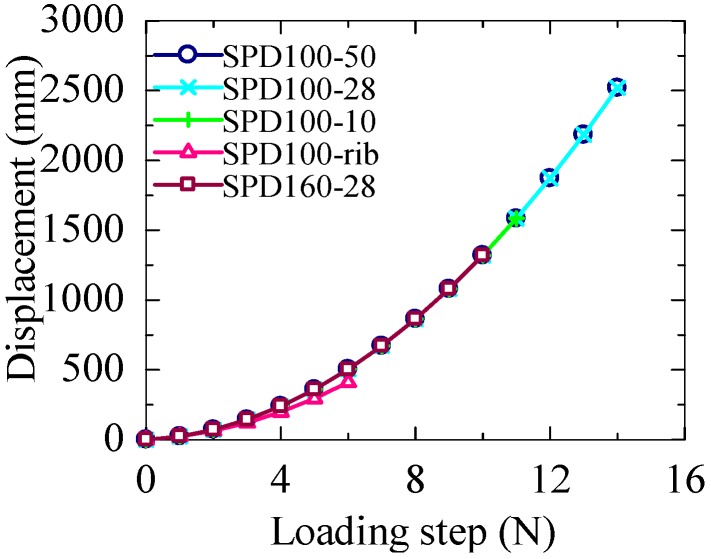
Cumulative displacement.

**Figure 8:**
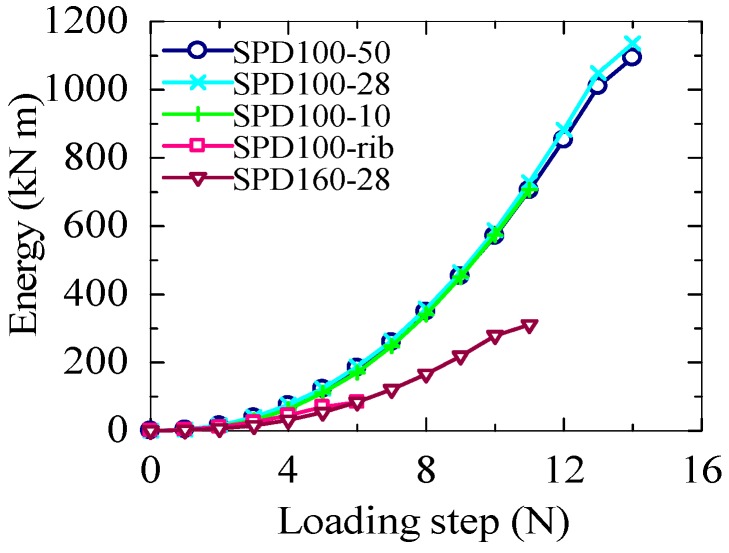
Cumulative energy.

The cumulative displacements and cumulative energies of the specimens are shown in Figure 7 and Figure 8, respectively. The loading histories of all the tests are almost the same; both SPD100-50 and SPD100-28 show the same ultimate displacement, 84 mm, which is the largest displacement observed among all the specimens. The ultimate displacement of SPD100-10 is 18 mm, which is smaller than that of SPD100-28. The smallest ultimate displacement is observed for the SPD100-rib damper, which is only around 35% of the maximum ultimate displacement. Simultaneously, the cumulative energy of the SPD100-rib damper is also lower than those of the other dampers. The ultimate displacement of SPD160-28 is 60 mm. Owing to the large shear area of the LYS100 dampers, their shear force is much larger than that of the SPD160-28 damper, which results in an increase in the energy absorption capacity of the LYS100 dampers.

**Figure 9:**
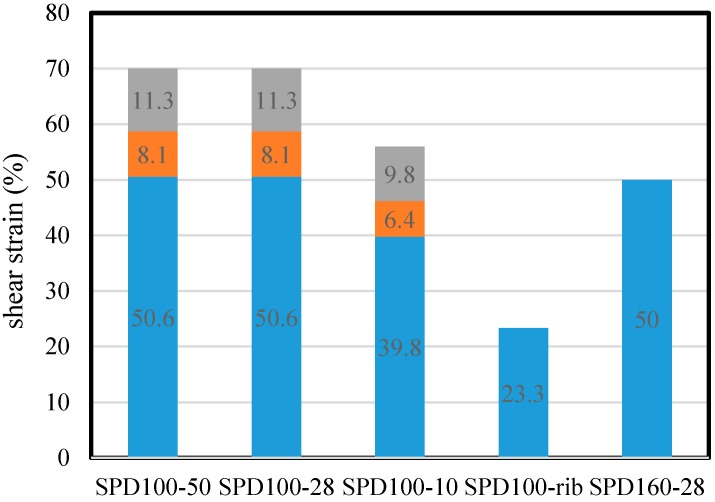
Ductility capacity.

The deformation capacities of the dampers are shown in Figure 9. Owing to the large transition arc of the dampers, the effective height of the shear panel is controversial [12]. The effective height of the dampers shown in Figure 6a–c is assumed to be 166 mm, which is the value obtained by summing up the panel height and the lengths of the two half-arcs. The effective heights of these dampers can also be calculated as 120 and 143 mm (panel height + 2 half-arc lengths), respectively. When the effective height of SPD100-50 and SPD100-28 increases, the corresponding ultimate shear strains of the three dampers are 70%, 58.7% and 50.1%, respectively. The ultimate shear strain of SPD100-10 is around 14%, which is smaller than that of SPD100-28. The smallest ultimate shear strain is only 23.3%, which is observed for SPD100-rib. Although the displacements of SPD100-50, SPD100-28, and SPD100-10 are much larger than that of SPD160-28, the differences in their maximum shear strains are not remarkable when the transition arcs are taken into account in the panel height.

#### 3.2.2. Failure Mode

**Figure 10:**
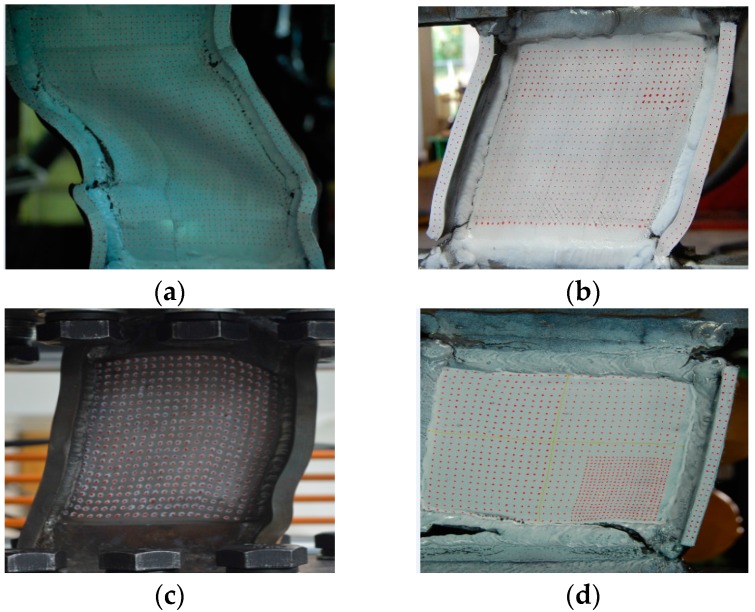
Failure modes of dampers: (**a**) SPD100-50; (**b**) SPD100-10; (**c**) SPD160-28; and (**d**) SPD100-rib.

The failure modes of these four specimens are shown in Figure 10. Deformation is observed in the arc area, which is connected to the shear panels and stiffeners. The height of the arc should be calculated as the effective height of the shear panel; the corresponding deformation capacity of SPD100-28 is calculated to be 50.6%. This value is similar to that of SPD160-28. The large arc height does not cause any significant improvement in the damper deformation capacity.

Stable in-plane plastic deformation is observed before the failure of the dampers. The failure process of SPD100-50 and SPD100-28 is the same. With an increase in the loading shear strain to around 40%, the appearance of a plastic fatigue crack of the rib in SPD100-50 is observed. This is followed by out-of-plane buckling of the shear panel. Unlike in the case of SPD100-50, the crack appears at the end of the ribs when the shear strain is 40%, which is 10% lower than the ultimate shear strains in SPD100-28. In SPD160-28, the appearance of the crack is delayed until the ultimate shear strain is reached. From the failure process of these specimens, it can be said that the geometry and material type of the ribs play important roles in out-of-plane buckling.

The SPD100-rib specimen and the other specimens are observed to have different failure modes. A small snap can be heard when the shear strain is 10%. A large crack can be observed when the shear strain is 20%; then, the dramatic damper force decreases until the damper undergoes failure. Over the entire test process, the panel shear deformation is not remarkable, and plastic interlayer sliding is not observed at all. This implies that brittle fracture is the main factor that leads to the failure of the SPD100-rib specimen, whereas ductile fracture is the failure mechanism of the remaining specimens.

### 3.3. Discussion on Lightweight Design

#### 3.3.1. Optimization of Boundary Constraints

(1) Stiffener

Although the stiffener heights of SPD100-50 and SPD100-28 are 50 mm and 28 mm, respectively, their ultimate shear strain and failure process are identical. When the stiffener height is reduced to 10 mm, the ultimate shear strain decreases considerably. This indicates that the optimal stiffener height is around two times the width of the heat-affected zone. In this case, overlap of the plastic hinge and the welding rib ends can be prevented effectively.

(2) Transition Arc

Under the same stiffener height, the connection arc heights of SPD100-28 and SPD160-28 are 23 mm and 2 mm, respectively. Their ultimate shear strains (50.6% and 50%, respectively) are also almost the same when the arc height is taken into account in the panel effective height. This implies that the use of a large connection arc of shear panels does not contribute to an improvement in the deformation capacity.

The difference between the side thickness values of the stiffener and the panel is no more than 3 mm in the case of the SPD160-28 specimen. The plastic deformation is concentrated in the central panel. This suggests that small shape mutations will cause large stress concentrations in large plastic deformations. This phenomenon is highly favorable for the lightweight design; a quantitative analysis of this phenomenon will be performed in future research. On the other hand, even if the arc is extremely small, stress concentration is not observed between the central panel and the stiffener. It can be inferred that shear stress, instead of diagonal tension stress, dominates the LYSPD under large plastic deformation. In this case, the transition arc has no contribution to the alleviation of the stress concentration, and it is a redundant design.

(3) Rib

The rib width of SPD160-28 reduces to 60 mm, which is 10 mm lower than that of SPD100-28. The strength change is enough to alleviate the stress concentration of SPD160-28 at the four panel corners. Owing the high deformation capacity of the LYS, the plastic hinge works effectively in conjunction with the ribs.

Not only should the stress concentration located at the four corners be restrained, but the interlayer deformation mechanism of the shear panel should also be maintained under cyclic loading by means of the ribs. Only in this case can the high deformation capacity of the damper be ensured. When the shear deformation of the panel is extremely large, the inclined deformation of the ribs is also large. Hence, this implies that a large elongation of the rib material is necessary. However, the ductility of Q235 is too low, which inhibits the interlayer shear deformation of the shear panel (see Figure 8d). The low ductility and high stiffness of the rib also cause the entire shear panel to be in an inclined tensile state. This leads to a huge increase in the stress concentration at the four corners and the damper cracks, even if the deformation is considerably small. The advantage of the high ductility of the LYS is negated by the Q235 ribs, which results in the maximum shear strain of SPD100-rib being smaller than half of that of the remaining four specimens. Therefore, Q235 is not a suitable material for the lightweight design of the LYSPD.

#### 3.3.2. Generality of Lightweight Design

SPD100-28 is made of LYS100, whereas SPD160-28 is made of LYS160. The elongation of the latter specimen is around 10% smaller than that of the former one. When the incremental shear strain is 5% in the hysteretic loading sequence, the maximum shear strains of the dampers of both LYS100 and LYS160 are identical to the elongations of the corresponding materials under uniaxial tension. Our experimental results suggest that the goal of lightweight design of the dampers can be achieved using LYS’s because of their high plastic deformation capacity.

#### 3.3.3. Shear Force and Lightweight Design

As shown in Figure 10, out-of-plane buckling is not observed in either SPD100-50 (as is the case with SPD100-28) or SPD160-28 before a crack appears in the panels. Both these specimens exhibit high deformation capacity. The maximum force of SPD100-50 (600 kN) is around 1.5 times that of SPD160-28 (400 kN), as shown in Figure 6. Hence, the damper force can be adjusted flexibly. When the damper response displacement is determined according to seismic design, the maximum allowable damper force can be adjusted by regulating the panel width. This method can be easily used in the lightweight design of the LYSPD, since the large bearing force can be provided simply by increasing the panel width by a small amount. Our experimental results show that the boundary constraints can provide a reliable guarantee of a lightweight damper. However, it should be noted that an appropriate adjustable range of the bearing force remains unclear owing to the limitations of the number of tests. The relation between the panel stability and the lightweight design will be discussed in detail in a subsequent work.

#### 3.3.4. Performance of the Lightweight Damper

The SPD100-50 damper is more beneficial for lightweight design than are conventional dampers. Meanwhile, around 47% of the weight of the SPD100-50 damper can be reduced further by optimization of the boundary constraints (SPD160-28). The accumulative plastic displacement and shear strain of the SPD160-28 damper are 1320 mm and 1100%, respectively. The corresponding damper force and dissipated energy are 40 t and 311 kN·m, respectively. It is surprising to note that this huge amount of energy can be dissipated by a small damper, which is only as large as around one half of an A4 paper in size. Both the goals of lightweight design and miniaturized damper design can be achieved simultaneously.

## 4. Conclusions

The lightweight design of a metallic damper is studied in this work. The mechanical properties of several metallic damping materials are systematically investigated under uniaxial tension and under shear by tests. The key findings of the study are summarized as follows.

1. LYS100 is found to exhibit superplastic deformation behavior under shear. The plastic properties of materials under shear cannot be simply predicted by the tensile stress–strain curves under uniaxial tension.
2. The ultimate uniaxial tensile strain of LYS160 is approximately 50%. However, the maximum shear strain of the LYSPD is more than 50% under the ±5% incremental reciprocal loading sequence. When the principal stress is consistent with the maximum energy consumption capacity of the damping structure, the lightweight design goal of the dampers can be accomplished.
3. Boundary constraints are important elements from the viewpoint of ensuring large deformation, and they dominate the adjustable force of the damper.
4. Both sufficient stiffness and high ductility of the boundary constraint components are necessary for ensuring large deformation and large energy consumption. Lightweight design of the dampers cannot be achieved by using boundary constraints made from low-ductility metallic damping materials such as ordinary carbon steel.
5. Optimal selection of the damping materials based on the maximum energy consumption criterion is beneficial for the lightweight design of the SPD. Although the density of aluminum is rather low, the maximum energy consumption per unit mass of Al6061 is only 1/6 times that of LYS100. LYS is the most optimal material among all the considered metallic damping materials for achieving the lightweight design goal of seismic dampers.

## Figures and Tables

**Table 1:** Specifications of test specimens.

No.	Name	Material	Diameter (mm)	Length (mm)	Test Type	
					Tension	Torsion
1	Q235	Ordinary carbon steel	10	100	Ten1	Tor1
2	Q235				Ten2	Tor2
3	Q235				Ten3	Tor3
4	AL6061	Aluminum 6061			Ten4	Tor4
5	AL6061				Ten5	Tor5
6	AL6061				Ten6	Tor6
7	SS316	Stainless steel 316			Ten7	Tor7
8	SS316				Ten8	Tor8
9	SS316				Ten9	Tor9
10	LYS225	Low-yield-strength steel 225			Ten10	Tor10
11	LYS225				Ten11	Tor11
12	LYS225				Ten12	Tor12
13	LYS160	Low-yield-strength steel 160			Ten13	Tor13
14	LYS160				Ten14	Tor14
15	LYS160				Ten15	Tor15
16	LYS100	Low-yield-strength steel 100			Ten16	Tor16
17	LYS100				Ten17	Tor17
18	LYS100				Ten18	Tor18
